# Contralateral Abdominal Pocketing in Salvation of Replanted Fingertips with Compromised Circulation

**DOI:** 10.1155/2014/548687

**Published:** 2014-10-14

**Authors:** Hyung-Sup Shim, Dong-Hwi Kim, Ho Kwon, Sung-No Jung

**Affiliations:** Department of Plastic and Reconstructive Surgery, College of Medicine, Uijeongbu St. Mary's Hospital, The Catholic University of Korea, 271 Cheonbo-ro, Uijeongbu-si 480-717, Republic of Korea

## Abstract

Abdominal pocketing is one of the most useful methods in salvation of compromised replanted fingertips. Abdominal pocketing has generally been performed in the ipsilateral lower abdominal quadrant, but we have also performed contralateral pocketing at our institute. To determine which approach is more beneficial, a total of 40 patients underwent an abdominal pocketing procedure in either the ipsilateral or contralateral lower abdominal quadrant after fingertip replantation. Dates of abdominal pocketing after initial replantation, detachment after abdominal pocketing, range of motion (ROM) before abdominal pocketing, and sequential ROM after the detachment operation and date of full ROM recovery and Disabilities of Arm, Shoulder, and Hand questionnaire (DASH) score were recorded through medical chart review. Mean detachment date, mean abduction of shoulder after the detachment operation, and mean days to return to full ROM were not significantly different between the ipsilateral and contralateral pocketing groups. However, the mean DASH score was significantly lower in the contralateral group than the ipsilateral group. There were also fewer postoperative wound complications in the contralateral group than in the ipsilateral group. We, therefore, recommend contralateral abdominal pocketing rather than ipsilateral abdominal pocketing to increase patient comfort and reduce pain and complications.

## 1. Introduction

In this era of supermicrosurgery for fingertip replantation, it is still difficult to achieve a high success rate in replantation of distal zone I amputations. Numerous methods have been proposed to increase the success rate; among these, the abdominal pocketing procedure is one of the most effective ways to salvage a compromised fingertip [1–3]. Abdominal pocketing has traditionally been performed on the ipsilateral side of the abdomen, but in this paper, we introduce contralateral abdominal pocketing.

## 2. Patients and Methods

**Figure 1:**
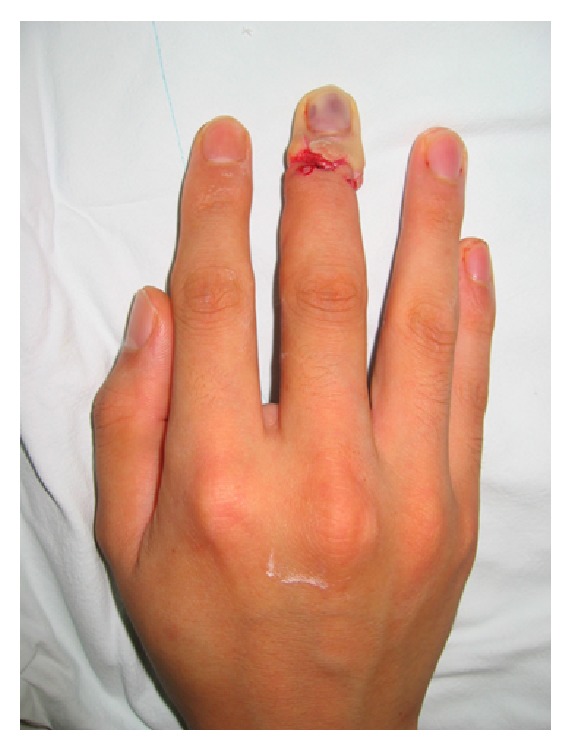
Replanted finger with vascular insufficiency.

All patients who underwent an abdominal pocketing procedure after replantation surgery for a zone I amputation of the finger between January 2004 and December 2012 at our center were included in this study. All electronic medical records were reviewed retrospectively, and the study was approved by the Institutional Review Board of Uijeongbu St. Mary's Hospital. Patients older than 20 years who underwent an ipsilateral or contralateral abdominal pocketing procedure after replantation of a single digit amputation distal to the lunula were included in this study, and replantation was performed with at least one arterial anastomosis. All patients who underwent abdominal pocketing had vascular compromise, which was defined as either vascular insufficiency (Figure 1) or vascular congestion. Exclusion criteria included patients who had trauma history of the same finger or range of motion (ROM) problems of the shoulder, elbow, or wrist joint. A total of 21 ipsilateral and 19 contralateral abdominal pocketing patients were finally included in our study. Date of abdominal pocketing after initial replantation, date of detachment after abdominal pocketing, ROM before abdominal pocketing, sequential ROM after the detachment operation, and date of full ROM recovery in external rotation and abduction of shoulder (90 degrees external rotation and 180 degrees abduction) were recorded after consultation with the rehabilitation medicine department. We also evaluated shoulder and hand function and associated pain using the Disabilities of Arm, Shoulder, and Hand questionnaire (DASH); a DASH score of 0 corresponds to optimal function and a score of 100 represents maximal disability [4]. DASH score was determined 4 days after the detachment operation. In addition, we compared the incidence of postoperative complications between the ipsilateral group (group I) and contralateral pocket group (group C).

## 3. Surgical Procedure

**Figure 2:**
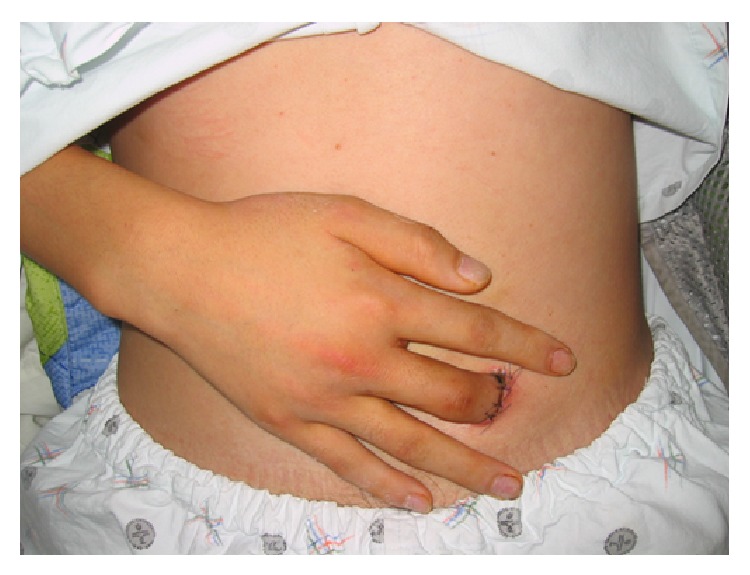
Patient with contralateral abdominal pocketing.

Under local anesthesia, we performed deepithelialization on the volar side of the replanted fingertip to prepare a contact area for the deep abdominal fascia. Then, we designed an incision line at the lower abdominal quadrant to put the finger in that would allow patients to flex their shoulder, elbow, and wrist naturally. We incised the skin and dissected subcutaneous tissue to reach the deep abdominal fascia, pocketed the prepared finger, and confirmed the contact of the deepithelialized fingertip with the deep abdominal fascia. After closure of subcutaneous tissue, abdominal skin and finger skin were closed together with nonabsorbable suture to prevent mobilization (Figure 2). Until the detachment operation, we utilized a cotton pad, an abdominal bandage, and an arm sling for immobilization, and the detachment operation was performed after an average of 18 days.

## 4. Results

**Figure 3:**
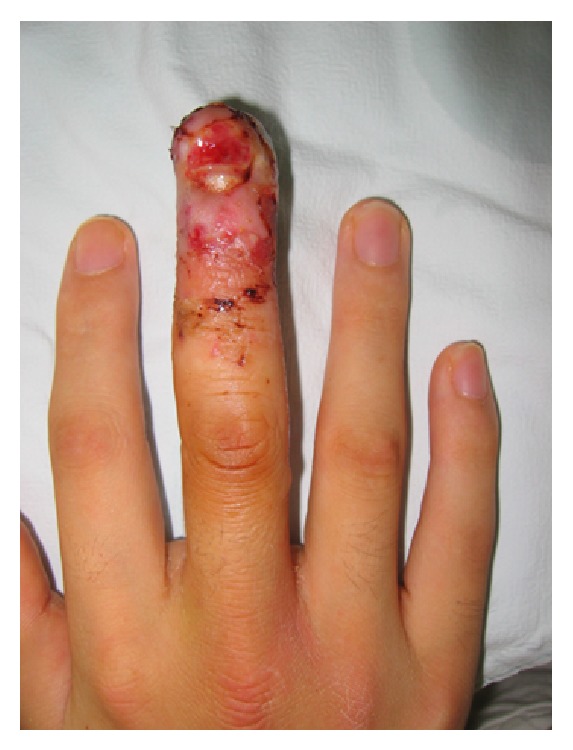
After 18 days of abdominal pocketing, the finger shown in Figure 1 survived completely.

Statistical analyses were conducted using SAS software version 9.3 (SAS institute, Cary, NC, USA) with an independent sample *t*-test, and *P* < 0.05 was considered significant. Demographic data of the patients in each group are presented in Table 1. A total of 40 patients were included in this study. Group I comprised 21 patients (15 males, 6 females) with a mean age of 43.5 years (range, 23–57 years), while group C comprised 19 patients (11 males, 8 females) with a mean age of 42.6 years (range, 25–55 years). There was no significant difference in patient age range between the two groups. Mean number of days to detachment was 18.48 ± 2.3 days in group I and 18.32 ± 2.5 days in group C, and mean degree of shoulder external rotation and abduction right after the detachment operation were 17.6 ± 4.6 and 107.4 ± 8.5 in group I, respectively, and 17.9 ± 3.0 and 105.5 ± 10.0 in group C, respectively. Mean days to return to full ROM of the shoulder after the detachment operation were 34.9 ± 7.9 days in group I and 31.7 ± 3.7 days in group C. There were no significant differences between groups. However, DASH scores were significantly lower (*P* = 0.03) in group C (59.4 ± 12.0) than group I (66.7 ± 9.0), indicating more functional and comfortable postoperative ROM in group C. Three cases of partial necrosis and two total losses of the fingertip occurred in group I, whereas only one case of partial necrosis that healed completely occurred in group C (Table 2, Figure 3).

## 5. Discussion

The lower abdomen has a good blood supply and redundant soft tissue and is also one of the best locations for distant flap procedures because of its proximity to the natural resting position of the arms and hands [5–13]. After the development of supermicrosurgery for replantation of fingertips, the lower abdomen began to be used as a pocketing area rather than as a distant flap in cases of vascular compromise following replantation. In fingertip replantation, an abdominal pocketing procedure is usually performed for patients with vascular compromise that is not resolved with medication, including antiplatelet agents [1, 2]. In our series, we implemented abdominal pocketing when vascular insufficiency or congestion was identified through a pinprick test after zone I amputation with one artery or one artery/one vein anastomosis, because reanastomosis is usually difficult to perform.

The majority of abdominal pocketing operations have been performed in the ipsilateral lower abdominal quadrant [1, 2]. However, at our institute, we have also used the contralateral side for abdominal pocketing and compared the outcomes of the two pocketing locations. First, contralateral pocketing provides more secure positioning than ipsilateral pocketing. In ipsilateral pocketing, the upper arm and forearm cannot rest against the abdomen and cannot be completely fixed in place even with the aid of arm slings and abdominal bandages; this increases the wound complication rate and can lead to severe complications such as accidental finger detachment. Contralateral pocketing, however, can reduce mobility with fixation of the shoulder and upper arm to the lateral side of the chest, and the center of the abdomen can even support the wrist and forearm, thereby providing greater stability. Second, due to the curvature of the abdomen, a great amount of flexion and extension of joints is required for close contact between the finger and deep fascia when using an ipsilateral pocketing approach, which results in needless tension not only in the joints of the hand and wrist, but also in the elbow and shoulder. In contrast, a contralateral pocketing approach increases the contact surface between a distal phalanx and deep fascia in a neutral and more comfortable arm and hand position. Third, there was no difference in initial postdetachment shoulder ROM between the ipsilateral and contralateral pocketing groups. Although our hypothesis was that it would take longer to recover full ROM after detachment with contralateral pocketing, this was not the case, as the comfortable positioning of the arm reduced the occurrence of symptoms resembling frozen shoulder, resulting in no significant difference in days till full ROM recovery between the two groups. With regard to evaluation of shoulder ROM, because the symptoms of patients in the postdetachment period were similar to those of frozen shoulder, we compared external rotation and abduction between groups. We also recorded elbow ROM during the study but did not describe these results because there were no differences between groups, including the degree of elbow flexion during the pocketing period. Most importantly, patients in the contralateral pocketing group had a lower mean DASH score, reflecting good ability to adapt, as well as satisfaction after the immediate detachment period, which indicates more rapid recovery of daily activity functions than patients in the ipsilateral pocketing group. This may be due to the less tension applied on the rotator cuff muscles, and further studies can be performed to reveal definite factors that lead to lower DASH scores. In addition to the comfort of patients, complication rates and the severity of the complications were lower in group C than in group I; however, there were too few complications to assess if this difference was statistically significant. Large-scale future studies are required to assess whether there are significant differences in complication rates according to ipsilateral or contralateral abdominal pocketing.

Although the contralateral abdomen has been used for distant flaps, no previous study has compared ipsilateral and contralateral abdominal pocketing. Our study can provide valuable information regarding the use of abdominal pocketing to save replanted fingertips with vascular compromise.

## 6. Conclusion

Abdominal pocketing is one of the most useful salvage methods for compromised fingertip replantations. We recommend contralateral abdominal pocketing rather than ipsilateral abdominal pocketing to increase patient comfort and reduce pain and complications.

## Figures and Tables

**Table 1:** Demographic data of patients included in the study.

	Ipsilateral group	Contralateral group
Total	21	19
Male	15	11
Female	6	8
Age (yr)	43.5 ± 9.6 (range 23~57)	42.6 ± 8.0 (range 25~55)

**Table 2:** Patient postoperation detachment date, external rotation, abduction, full ROM recovery time, and DASH score.

	Ipsilateral group	Contralateral group
Detachment date (POD) (mean ± SD)	18.48 ± 2.3	18.32 ± 2.5
External rotation (mean ± SD)	17.6 ± 4.6	17.9 ± 3.0
Abduction (mean ± SD)	107.4 ± 8.5	105.5 ± 10.0
Full ROM recovery date (mean ± SD)	34.9 ± 7.9	31.7 ± 3.7
DASH (mean ± SD)	66.7 ± 9.00	59.4 ± 12.0
